# Characteristics of Moyamoya Disease in the Older Population: Is It Possible to Define a Typical Presentation and Optimal Therapeutical Management?

**DOI:** 10.3390/jcm10112287

**Published:** 2021-05-25

**Authors:** Ignazio G. Vetrano, Anna Bersano, Isabella Canavero, Francesco Restelli, Gabriella Raccuia, Elisa F. Ciceri, Giuseppe Faragò, Andrea Gioppo, Morgan Broggi, Marco Schiariti, Laura Gatti, Paolo Ferroli, Francesco Acerbi

**Affiliations:** 1 Department of Neurosurgery, Fondazione IRCCS Istituto Neurologico Carlo Besta, 20133 Milan, Italy; francesco.restelli91@gmail.com (F.R.); gabriellaracc@gmail.com (G.R.); morgan.broggi@istituto-besta.it (M.B.); marco.schiariti@istituto-besta.it (M.S.); paolo.ferroli@istituto-besta.it (P.F.); francesco.acerbi@istituto-besta.it (F.A.); 2 Cerebrovascular Unit, Fondazione IRCCS Istituto Neurologico Carlo Besta, 20133 Milan, Italy; anna.bersano@istituto-besta.it (A.B.); isabella.canavero@istituto-besta.it (I.C.); 3 Interventional Neuroradiology Unit, Fondazione IRCCS Istituto Neurologico Carlo Besta, 20133 Milan, Italy; elisa.ciceri@istituto-besta.it (E.F.C.); giuseppe.farago@istituto-besta.it (G.F.); andrea.gioppo@istituto-besta.it (A.G.); 4 Cellular Neurobiology Laboratory, Fondazione IRCCS Istituto Neurologico Carlo Besta, 20133 Milan, Italy; laura.gatti@istituto-besta.it; 5 Experimental Microsurgical Laboratory, Fondazione IRCCS Istituto Neurologico Carlo Besta, 20133 Milan, Italy

**Keywords:** cerebral angiopathy, elderly, extracranial-intracranial bypass, internal carotid artery, moyamoya, middle cerebral artery, revascularization, superficial temporal artery

## Abstract

Whereas several studies have been so far presented about the surgical outcomes in terms of mortality and perioperative complications for elderly patients submitted to neurosurgical treatments, the management of elderly moyamoya patients is unclear. This review aims to explore the available data about the clinical manifestation, characteristics, and outcome after surgery of older patients with moyamoya arteriopathy (MA). We found only two articles strictly concerning elderly patients with MA. We have also evaluated other reported adult series of moyamoya patients, including elderly cases in their analysis. Patients with MA above 50 years old may be considered a peculiar subset in which patients are often presenting with ischemic symptoms and a higher Suzuki grade. Conservative treatment may be proposed in asymptomatic or stable cases due to their fragility and possible increase of post-operative complications, while the best surgical options in symptomatic cases are still under investigation, although we believe that a minimal invasive superficial temporal artery—middle cerebral artery bypass could be considered the treatment of choice for the immediate effect on brain perfusion with a limited rate of post-operative complications.

## 1. Introduction

Moyamoya angiopathy (MA) is an uncommon cerebrovascular disorder, characterized by progressive occlusion of the supraclinoid internal carotid artery (ICA) and its main branches within the circle of Willis. The ICA stenosis leads to the formation of an aberrant, compensatory, fine vascular network that on angiograms looks like a puff of cigarette smoke (“moyamoya” in Japanese) [1,2]. The pathogenesis of idiopathic MA is unknown. Anomalies in angiogenesis, due to the detection of increased cytokine and growth factor concentrations have been invoked as potential disease mechanisms [3]. Given the familial rate, especially in the Eastern countries, and the ethnic differences, genetic factors are also believed to be involved [4,5]. In Japan, the estimated prevalence and incidence rates are 3.16 and 0.35 per 100,000 persons, with a female predominance. In Europe, the prevalence is probably 10 times lower than in Asia [6,7,8], whereas it is increasingly diagnosed in ischemic or hemorrhagic stroke, or headache patients undergoing neuroradiological examinations.

In addition to the idiopathic variant, MA can also arise secondary to cranial therapeutic radiation or in association with other conditions, including chromosomopathies, sickle cell disease, or neurofibromatosis, in a condition called moyamoya syndrome (MMS), distinguishing it etiologically from idiopathic moyamoya disease (MMD). Finally, MA occurring unilaterally has been recently defined as a separate entity [4].

MA has several unique features, which include (at least in Asia) two peaks of age distribution during the first and fourth decades of life [1,9,10]. Reports on North American patients, instead, have shown a unimodal age distribution with a peak during the third and fourth decades of life [11]. The clinical manifestations are slightly different according to the age of onset. Ischemic attacks are more frequent in the pediatric population [1,9,12], whereas adult patients can have ischemic attacks, intracranial bleeding, or both. However, age influences not only the presentation of MA but also the treatment outcomes [13,14,15].

The surgical treatment is usually based on direct and indirect revascularization, aiming at improving cerebral hemodynamics and decreasing the pathological collateral network development [16], thus reducing the risk of further ischemic events; furthermore, direct bypass seems to prevent also re-hemorrhage in adult moyamoya cases with a hemorrhagic presentation [17]. However, the natural history of the disease and the effect of surgical treatment in the older population of moyamoya patients is not clear. As a matter of fact, in the last few decades, the world population has progressively become older. Increased life expectance, due to improved socio-economic and health conditions, is associated with global population aging, which implies a progressive surge in health demand. In 2018, nearly one-fifth of the European Union population was aged 65 years old and more [18,19,20]. With a larger portion of the population aging, the demand for medical and surgical treatment has increased too [21]. Whereas several studies have been so far presented about the surgical outcome in terms of mortality and perioperative complications for elderly patients submitted to neurosurgical treatments [22,23,24,25], the existing literature about this issue in MA is really limited. This could be secondary to the peculiar characteristics of the bimodal age distribution, with the second peak around 40 years, at least in the Asian population. Moreover, the prevalence of asymptomatic patients with MA may be higher than previously thought, although the development of noninvasive neuroradiological examination has increased the opportunity to identify this subpopulation of patients who have experienced no stroke episodes [26]. Therefore, such patients can overcome the “adult” peak of clinical onset, leading to a delay of the diagnosis.

This review aims to explore the available data about the clinical manifestation, characteristics, and outcome after surgery of older patients with MA, to try answering specific questions: which MA patients could be considered as “elderly”, and how to manage them?

## 2. Materials and Methods

We performed a search on MEDLINE and Scopus databases for the indication terms: surg* OR neurosurg*OR treatment*AND Moyamoya AND elderl* OR old*, along with its derivatives, with the corresponding MESH (Medical Subject Heading) terms. The search was conducted on the literature before January 2021, comprising only articles with English full text, without historical limitation, although it did not strictly follow the criteria for a systematic review. The reference lists of relevant papers were inspected for further studies that could fit the inclusion criteria. The lack of an exact definition of elderly for MA, however, led us to analyze studies specifically concerning the treatment and the surgical outcome of adult patients, to identify possible elderly patients, even if they were not clearly stated in the title or the abstract. We arbitrarily considered as elderly, in this analysis, patients older than 50 years. Considering the extreme heterogeneity and the limited number of available series, that excluded the possibility to strictly and adequately perform a systematic review, we present the data as a comprehensive (narrative) review.

## 3. Results

We found only two articles strictly concerning elderly patients with moyamoya arteriopathy [27,28]. Furthermore, we have also evaluated other reported adult series of moyamoya patients, including elderly cases in their analysis [26,29,30,31].

### 3.1. Demographic Details

Gupta et al., in 2016, presented eight patients with a median age of 63 years, ranging from 60 to 71 years [27]. Six patients were females, and only two were males; the majority of patients were Caucasians. Four patients were diagnosed with bilateral moyamoya while the others had unilateral disease, for a total of 12 hemispheres. The presentation was hemorrhagic in two patients and ischemic in the other six. Median mRS was 1, with all patients presenting with a score of either 1 or 2. A median Suzuki stage of 4 was found in their cases.

Ge et al. reported, in 2017, on a series of 87 Chinese patients, for a total of 164 hemispheres, with a mean age at diagnosis of 54 years (range 50–67 years) [28]. Interestingly, this series lacked the usually reported female predominance, with a ratio of female-to-male patients of 1:1. Only two patients (2.3%) had a familial history, whereas 46 (52.8%) had a history of hypertension, and 16 (18.4%) presented hyperlipidemia. Ischemia was the presenting symptom in the majority of patients (58 out of 87 patients, 66.6%), while hemorrhage was present in 33.3% (29 cases). An initial mRS > 2 was reported in 18 out of 87 cases (20.7%). Most patients showed a Suzuki angiographic stage of 4 or 5 (51.2%); 14 patients (16.1%) patients had unilateral disease. The authors also reported involvements of the posterior cerebral artery in 25.3% of the cases.

Other authors included older patients in articles not specifically addressing this subset of population. Kuroda and coauthors presented, in 2007, a multicentric Japanese study involving 40 asymptomatic patients later diagnosed as affected by MA [26]. This series included 13 males and 27 females, with a mean age at diagnosis of 41.4 ± 12.6 years, ranging from 13 to 67 years, for a total of 77 hemispheres. Among the entire series, nine patients were 50–59 years old, and two in the decade 60–69; however, detailed clinical characteristics were available only for patients developing cerebrovascular events or showing silent radiological changes in the follow-up. In this subgroup of patients, 4 out of 11 cases were older than 50 years old (three females, one male), two of them developing cerebrovascular events (one stroke and one hemorrhage), and two showing silent radiological progression.

In addition, Larson et al. presented a retrospective series of 180 patients with MMD and MMS, aiming to evaluate the prevalence of pro-thrombotic states among patients affected by MA [29]. Among the entire series, three patients with MMD, and five with MMS were older than 60 years, and one of them (with MMD) presented a pro-thrombotic condition. No other information was available regarding the clinical condition in this subgroup of patients. Kuroda et al. evaluated the clinical course of conservatively managed moyamoya patients, as a 62 years-old man with bilateral disease, presenting, at 60 months follow-up, with an ischemic stroke, characterized by cerebral infarction in the left hemisphere, and radiological disease progression bilaterally [30]. Moreover, hemorrhagic stroke, disease progression, or microbleeding were also diagnosed in three patients over 50 years old (two patients of 51 years and one of 56). These studies, however, could provide only indirect and partial information about clinical manifestation and therapeutic management of this specific subgroup of patients [30] (Table 1).

### 3.2. Treatments and Outcome

In the Gupta et al. series, all patients presented clinical events related to moyamoya arteriopathy; therefore, in all of them a surgical indication was proposed, but only six patients underwent surgical treatment because two of them refused a surgical indication [27]. One of these patients necessitated a bilateral intervention, thus a total of seven hemispheres were treated. Both patients who did not undergo surgical interventions presented repeated cerebrovascular events in the follow-up. Among patients submitted to a surgical procedure, the majority underwent an indirect revascularization (EDAS in four patients and five hemispheres) while direct revascularization was performed only in two cases. Post-operative angiography demonstrated in five patients a Matshushima grade of B or C, indicating perfusion of up two-thirds of the MCA territory. Immediately post-operatively one patient has an improved mRS, three were stable and two worsened their pre-op mRS. During a median postoperative follow-up of 14.5 months, one patient submitted to bilateral EDAS suffered from bilateral acute infarction one week after surgery, while one patient died 6 months after surgery for an unrelated cause.

In the Ge and coauthors’ series, 74 patients underwent revascularization surgery, while 13 patients were conservatively treated [28]. Of the 74 patients operated on, 13 had bilateral surgery, for a total of 87 procedures. Of these, the majority (70%, 60 surgeries) was represented by direct revascularization (STA-MCA bypass). A total of 20 hemispheres were submitted to encephaloduromiosinangiosis and seven to multiple burr holes. Regarding postoperative complications, they were reported in 23 out of 87 surgeries (26.4%) before hospital discharge: in five cases (6.8% per patient, 5.7% per operation), patients experienced a post-operative infarction within 48 h after surgery; one patient (1.35% per patient, 1.14% per operation) presented a hemorrhagic stroke post-operatively; 10 patients experienced new neurologic deficits before discharge; four patients had wound infection and three had clinically relevant hyperperfusion syndrome. Multivariate analysis showed that the presence of diabetes was a predictor of an adverse event after surgery. At long-term follow-up, after a mean period of 35.5 ± 22.2 months, 9 out of 74 patients in the treated group (12.2%) presented a new cerebrovascular event (6 new hemorrhages and 3 new ischemia), while 3 out of 13 patients in the conservatively treated group (23.1%) presented a new cerebrovascular event (two hemorrhages and one ischemia). This difference was not statistically significant. On patients submitted to brain perfusion studies (72 cases), an improvement of cerebral perfusion was found in 56.7% of the surgically treated patients (34 out of 60), compared to 16.7% of the conservatively managed patients (2 out of 12). However, no significant difference in mRS score between surgically and conservatively treated patients was found at the latest follow-up. Long-term angiographic data were not available in this series. 

Hyun et al. analyzed a series of 165 surgically-treated adult MA patients (mean age 36 years, range 20–62 years), for a total of 246 revascularization procedures, to evaluate possible prognostic factors associated with perioperative ischemic complications in adult-onset MA [31]. Among the series, they noticed a 59 years-old female with a Suzuki stage of 3 and multiple preoperative ischemic episodes who presented right temporoparietal ischemia after indirect-only revascularization, which determined a worsening of mRS score. Despite the single case, in the entire series of adult patients, preoperative multiple ischemic episodes were associated with perioperative ischemic complications. In a wide series of 470 Chinese patients, Bao et al. found that older age at symptom onset, posterior circulation involvement, and the presence of multiple ischemic attacks were predictors of postoperative or subsequent strokes [13]. Nonetheless, only about 2% of patients were between 60 and 69 years old, and more specific data were not available; globally, surgical revascularization determined a decrease of subsequent strokes’ incidence, with a preserved functional independence in the majority of patients included.

## 4. Discussion

The natural course of “elderly” MA patients is still unclear, as our narrative review shows. Despite the very limited number of literature reports strictly focusing on this topic, it could be hypothesized that with the increased application, in clinical routine practice, of non-invasive diagnostic methods (as MRI and MRA), the incidence of asymptomatic moyamoya disease in the future year will be higher than previously thought. The accessibility of neuroradiological imaging has determined an increase of brain lesions, even if asymptomatic. Vermeer et al. analyzed the cerebral MRI scans obtained from 1995 to 1996 of 1077 elderly patients aged 60 to 90 years (mean, 72 years) without a history of stroke or TIA [32]. They found that patients harboring silent brain infarcts and white matter lesions had a five-times higher stroke incidence than those without, and this strongly increased risk could not be explained by the classical, major stroke risk factors, and are independent of the latter. Considering the technological level of MRI more than 20 years ago, one could postulate that, at least in a little part, some of that patients should be affected by a steno-occlusive ICA disease, maybe compatible with MA. 

### 4.1. Which Moyamoya Patients Are “Elderly” and What Are Their Characteristics? 

One of the most interesting results from literature analysis is represented by the uncertainty on who is considered “elderly” in moyamoya.

In the first specific report dedicated only to elderly moyamoya patients by Gupta et al. [27], the authors defined as “elderly” patients who were 60 years or older at the time of surgery. This small but homogeneous series by a North-America center included mainly Caucasian patients, with a female predominance (3:1 ratio), supporting the not-Asian pattern of MA presentation (unimodal peak of age, greater propensity towards females). The mean angiographic stage was 4, and the ischemia-related conditions were predominant. These findings were similar also in a subsequent, bigger series of Ge and coauthors [28]. The 87 Chinese patients included presented a mean age of 54 years, ranging from 50 to 67 years. Differently from other Asiatic series, this one lacked female predominance, with a female-to-male ratio of 1:1. Additionally in this series, nevertheless, ischemia (mainly infarction) was the most common presenting symptom, and the transient ischemic attack was the second most common presentation. Familiarity or classic risk factors for stroke as hypertension and hyperlipidemia were presented only in a minority of cases. As for the Gupta’s series, the majority of patients showed a Suzuki stage of 4 or 5. 

Excluding the above-mentioned specific report on elderly patients, the other series analyzed in the present review show some common characteristics [26,29], whereas it is difficult to disaggregate some data. However, in all series, there is a minority of patients comprised among 50 and 67 years [26], or over 60 [29], that present, also if asymptomatic, an advanced Suzuki stage at diagnosis, with a prevalent ischemic pattern (both symptomatic or not). Thus, based on the few specific cases available in the literature, we could consider that “older” moyamoya patients are above 50 years old, without family history, with vascular risk factors, usually presenting with ischemic symptoms, a more advanced angiographic stage (usually at least Suzuki stage of 4), and a lower involvement of PCA; in addition, in this subset of patients, the female predominance becomes less evident. However, data are limited and could become the object of future, prospective studies, specifically evaluating the clinical and socio-demographic characteristics of this subset of patients. 

### 4.2. What Is the Best Management? Does Age Have an Impact on the Outcome?

Data about natural history in moyamoya patients older than 50 years old are rather poor. According to the specific studies considered in this review, it is not possible to ascertain the clinical course of the disease in all the cases. Specifically, although all the 87 elderly patients in the study by Ge and coauthors [28] presented a cerebrovascular event as the initial symptom (either ischemic or hemorrhagic), the progression of symptoms was not reported in their series. In addition, it was not clear why a conservative treatment was proposed in 13 out of 87 patients. Nonetheless, in these conservatively managed cases, the risk of a new cerebrovascular event (mostly hemorrhagic) was around 23%, higher than the risk of a new event in the treated group, although this difference did not reach a statistical significance. However, in patients submitted to perfusion studies, a low percentage of cases (16.7%) seems to improve their brain perfusion even without any specific treatment. More interesting results about the natural history could be derived from the study by Kuroda and coauthors that showed a clinical or radiological progression in 36% of the initial elderly asymptomatic patients [26]. This clinical progression seems to be even higher if the patient was initially symptomatic, as reported by Gupta et al. in their series, where five out of eight cases had progressive deterioration requiring surgical intervention [27]. Thus, according to the very limited data available in the literature, there is a variable but consistent risk of ischemic or hemorrhagic progression in a patient with moyamoya arteriopathy older than 50 years old, even if initially asymptomatic. Thus, implementing only a conservative treatment strategy based mostly on antiplatelet therapy could be not ideal for every elderly patient. 

At the moment, instead, surgical revascularization, including both direct bypass mainly by STA-MCA microanastomosis, and multiple types of indirect revascularization, alone or in combination, represent the primary treatment for moyamoya arteriopathy, in the pediatric and adult population [13,14,17,31,33,34]. A debate exists in the literature regarding the best of these options, with studies supporting the use of direct bypass [35,36,37,38,39,40], and others favoring indirect revascularization [41,42,43,44,45], or a combination of both [46,47]. However, safety and efficacy at short-term and long-term follow-up of different surgical strategies in elderly patients have not been established yet.

Indirect revascularization procedures have been considered more effective for the pediatric population than for adult cases [33,44,48,49,50,51]. In particular, this seems to be related to the production of certain angiogenic factors that decrease with age [49,52]. The post-operative collateral formation through indirect bypass surgery has been reported to be moderate or poor in adult patients older than 40 years old submitted to combined procedures, while in the same group the effect of direct revascularization is increased [49]. 

Therefore, due to the different clinical responses to direct versus indirect surgical interventions in pediatric and adult populations [15], age-specific treatment paradigms have been suggested. This is particularly true in the case of elderly moyamoya patients, also considering that safety, efficacy, and clinical outcomes in elderly patients are unclear. 

Multiple factors should be considered before deciding when to perform a revascularization procedure in such patients and which kind of procedure should be preferred. Firstly, one pivotal element to consider, in this specific subset of patients, is to be sure that the patient has a real moyamoya arteriopathy and not intracranial atherosclerotic stenosis or occlusion [53], that are unlikely to respond to indirect bypass procedures [27]. This specific issue is highlighted also by Ge et al., which suggested performing high-resolution MRI to differentiate MMD from moyamoya syndrome due to atherosclerosis “if a patient had 2 or more risk factors of atherosclerosis” [53]. Moreover, the intrinsic frailty secondary to the increasing age, coupled with the reduced capillary densities and decreased rates of angiogenesis in response to ischemia and infarcts [52], could affect the possible response to the indirect bypass procedures, which may not provide the same benefits to elderly patients as in the younger adults, as pointed out by Gupta et al. [27]. Additionally, the role of intracranial collateral status, which appears to be correlated with the severity of the clinical symptoms and the therapeutic prognosis in younger patients [54], can affect the outcome in elderly moyamoya, despite the lack of specific studies on this subgroup of patients. Again, the co-existence of multiple, systemic medical issues, that increases according to age, could affect the outcome also more than the patient’s specific cerebrovascular condition. For example, diabetes seems to represent a risk factor for postoperative events in elderly patients, at least in the white and African-American moyamoya population [34]. The rate of postoperative adverse events in the unique large series of elderly patients that has been published is around 26%, including 6.9% of new post-operative cerebrovascular events and 11.5% of new neurological deficits [28]. Although in this series a direct bypass was the most prevalent type of procedure, there was not specified if these adverse events were more common in patients submitted to indirect revascularization only [28]. In the study by Gupta et al. one out of the six treated patients, whose treatment consisted of indirect revascularization, presented post-operative infarcts one week after surgery [27]. As elderly patients may be more prone to the hemodynamic stress of general anesthesia and surgical procedure than younger patients, the fact that indirect revascularization takes a longer period to improve perfusion, and that this aspect is also questioned at long term follow-up in elderly patients [49], could make a minimal invasive STA-MCA bypass procedure, similar to the one that has been proposed also in atherosclerotic patients [55], the treatment of choice for elderly symptomatic patients. 

## 5. Conclusions

The natural history of elderly patients with moyamoya disease is still uncertain. Patients with moyamoya disease above 50 years old may be considered a peculiar subset in which patients are often presenting with ischemic symptoms and a higher Sukuzi grade. The optimal management of these patients is also unclear: a conservative treatment may be proposed in asymptomatic or stable cases due to their fragility and possible increase of post-operative complications, while the best surgical options in symptomatic cases are still under investigation, although we believe that a minimal invasive STA-MCA bypass could be considered the treatment of choice for the immediate effect on brain perfusion with a limited rate of post-operative complications. Further studies with larger series are needed to better investigate this subset of patients with moyamoya arteriopathy.

## Figures and Tables

**Table 1:** Summary of the surgical series included in the review.

First Author	Number of Patients	Age	Ethnicity	Presentation	Hemispheres	Suzuki Grade
Gupta [27]	Eight	60–71 years (median 63)	6 Caucasians, 1 African American 1 Asian	Six ischemic Two hemorragic	12	4 (median)
Ge [28]	87	50–67 years (mean 54)	Asian	58 (66.6%) ischemic 29 (33.3%) hemorragic	164	4 or 5 in 51.2%
Kuroda [26]	11/40	9 were 50–59 years old, 2 n the decade 60–69	Asian	Asymptomatic	77 (entire series)	75% of the hemispheres were stage 3 or stage 4; older patients had significantly more advanced stage
Larson [29]	8/180	>60 years	79% Caucasians	n.a.	n.a.	n.a.
